# Osteopontin deficiency reduces kidney damage from hypercholesterolemia in Apolipoprotein E-deficient mice

**DOI:** 10.1038/srep28882

**Published:** 2016-06-29

**Authors:** Zouwei Pei, Takafumi Okura, Tomoaki Nagao, Daijiro Enomoto, Masayoshi Kukida, Akiko Tanino, Ken-ichi Miyoshi, Mie Kurata, Jitsuo Higaki

**Affiliations:** 1 Department of Cardiology, Pulmonology, Hypertension and Nephrology, Ehime University Graduate School of Medicine, Ehime, Japan; 2 Department of Pathology, Ehime University Proteo-Science Center and Graduate School of Medicine, Ehime, Japan

## Abstract

Hypercholesterolemia is a well-established risk factor for kidney injury, which can lead to chronic kidney disease (CKD). Osteopontin (OPN) has been implicated in the pathology of several renal conditions. This study was to evaluate the effects of OPN on hypercholesterolemia induced renal dysfunction. Eight-week-old male mice were divided into 4 groups: apolipoprotein E knockout (ApoE^*−/−*^) and ApoE/OPN knockout (ApoE^*−/−*^/OPN^*−/−*^) mice fed a normal diet (ND) or high cholesterol diet (HD). After 4 weeks, Periodic acid-Schiff (PAS) and oil red O staining revealed excessive lipid deposition in the glomeruli of ApoE^*−/−*^HD mice, however, significantly suppressed in ApoE^*−/−*^/OPN^*−/−*^HD mice. Lectin-like oxidized low-density lipoprotein receptor-1 (LOX-1) expression was lower in the glomeruli of ApoE^*−/−*^/OPN^*−/−*^HD mice than ApoE^*−/−*^HD mice. *In vitro* study, primary mesangial cells were incubated with recombinant mouse OPN (rmOPN). RmOPN induced LOX-1 mRNA and protein expression in primary mesangial cells. Pre-treatment with an ERK inhibitor suppressed the LOX-1 gene expression induced by rmOPN. These results indicate that OPN contributes to kidney damage in hypercholesterolemia and suggest that inhibition of OPN may provide a potential therapeutic target for the prevention of hypercholesterolemia.

ApoE^*−/−*^ mice are considered a well-accepted model of hypercholesterolemia^1^. In apoE^*−/−*^ mice, dyslipidemia-related kidney injury is associated with marked pathological alterations, including lipid deposition in the glomerulus, mesangial expansion, and increased extracellular matrix (ECM) area^2^,^3^. Increasing evidence has shown that lipid accumulation in the kidney contributes to the progression of chronic kidney disease (CKD)^4^,^5^. However, the underlying pathophysiological mechanisms of the relationship between hypercholesterolemia and renal injury are not yet fully understood.

Lectin-like oxidized low-density lipoprotein receptor-1 (LOX-1) is a receptor for oxidized low-density lipoprotein (ox-LDL). LOX-1 binds to multiple ligands, has diverse physiological functions, and plays a critical role in signal transduction. It may be a key factor in the development of hypertension, diabetes mellitus, and hyperlipidemia^6^,^7^,^8^.

Osteopontin (OPN) is a secreted glycoprotein that is found in many organs; bone and kidney show the greatest OPN content^9^. In addition, most epithelial lining cells also express OPN constitutively, and OPN is found in many secretions, including urine, saliva, milk, and bile^10^. The binding of OPN to integrin receptors through the highly conserved RGD motif promotes cell adhesion, chemotaxis, and signal transduction in various cell types^11^. OPN is thought to play a role in the renal damage associated with inflammatory glomerulonephritis, obstructive uropathy, and tubule interstitial disease^12^. One of our recent studies showed that OPN deficiency protects against aldosterone-induced inflammation, oxidative stress, and interstitial fibrosis in the kidney^13^. However, the function of OPN in hypercholesterolemia-induced renal injury is not clear.

## Results

### *In vivo* studies

#### Metabolic characteristics

The metabolic characteristics of ApoE^*−/−*^ mice and ApoE^*−/−*^/OPN^*−/−*^ mice after 4 weeks of dietary treatment (high-cholesterol diet: HD, or normal diet: ND) are summarized in Table 1. Two-way ANOVA was applied to analyze the effects of gene (ApoE^*−/−*^ and ApoE^*−/−*^/OPN^*−/−*^) and diet (ND and HD). HD markedly increased total cholesterol (TC) and low-density lipoprotein-cholesterol (LDL-C) in ApoE^*−/−*^ mice and ApoE^*−/−*^/OPN^*−/−*^ mice. However, interestingly, TC and LDL-C were lower in ApoE^*−/−*^/OPN^*−/−*^ mice than in ApoE^*−/−*^ mice with both ND and HD treatment. Triglycerides (TG) in ApoE^*−/−*^ mice and ApoE^*−/−*^/OPN^*−/−*^ mice treated with HD were lower than in ApoE^*−/−*^ mice and ApoE^*−/−*^/OPN^*−/−*^ mice treated with ND, respectively. Body weights and kidney weights did not differ among the four groups. Renal function and blood urea nitrogen (BUN) also did not differ among the four groups.

#### OPN concentration in plasma was significantly increased in ApoE^*−/−*^ mice fed HD

**Figure 1:**
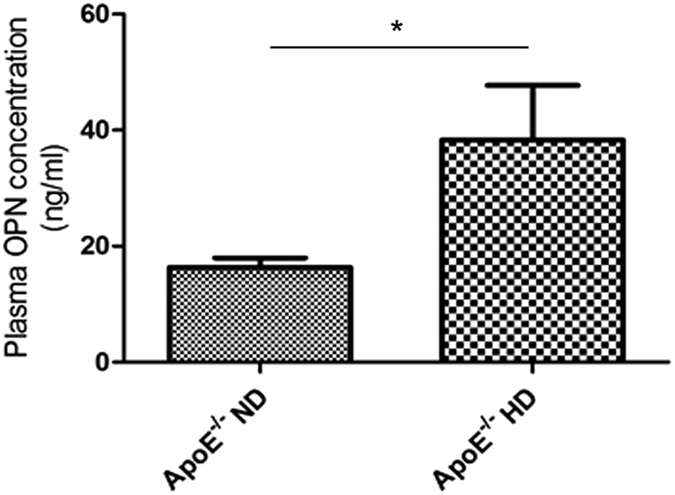
Plasma OPN concentration in ApoE^−/−^ND and ApoE^−/−^HD mice. Plasma OPN concentration was measured by ELISA in ApoE^*−/−*^ mice after 4 weeks of dietary treatment. Data are means ± SEM; n = 5 per each group. *P < 0.001.

We measured plasma OPN concentrations in ApoE^*−/−*^ mice. OPN concentration was significantly increased in ApoE^*−/−*^ mice after 4-week treatment with HD (ApoE^*−/−*^HD mice) (Fig. 1).

#### OPN deficiency reduced glomerular lipid accumulation in HD mice

**Figure 2:**
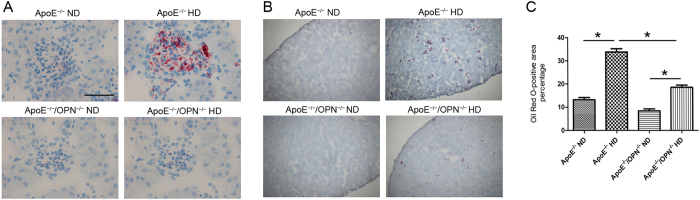
Renal lipid accumulation in the four groups after 4weeks of dietary treatment. (**A**) Representative oil red O staining in the kidneys. Red: oil red O-positive cells; blue: hematoxylin counterstaining. Scale bar = 50 μm. (**B**) Representative oil Red O staining in the kidneys. Red: oil red O-positive cells; blue: hematoxylin counterstaining. Scale bar = 500 μm. (**C**) Bar graph showing oil red O-positive cells in the kidney. Data are means ± SEM; n = 4 in each group. *P < 0.001. Two-way ANOVA, gene, p < 0.001; diet, p < 0.001; gene × diet, p < 0.001.

We used oil red O staining to evaluate glomerular lipid accumulation. We detected increased lipid retention in glomeruli of ApoE^*−/−*^HD mice. Interestingly, ApoE^*−/−*^/OPN^*−/−*^ mice showed markedly reduced glomerular lipid deposition compared with ApoE^*−/−*^ mice despite consumption of HD (ApoE^*−/−*^/OPN^*−/−*^HD mice) (Fig. 2A,B). Glomerular volume and lipid accumulation were quantified by scanning 10 non-overlapping glomeruli from each kidney section stained with oil red O. Two-way ANOVA showed the effects of gene and diet on the glomerular lipid accumulation (gene, p < 0.001; diet, p < 0.001; gene × diet, p < 0.001). Post-hoc analysis leveled that glomerular area contained lipids in ApoE^*−/−*^HD mouse was reduced in ApoE^*−/−*^/OPN^*−/−*^HD mice (p < 0.001) (Fig. 2C).

#### OPN deficiency reduced renal triglyceride concentration in HD mice

**Figure 3:**
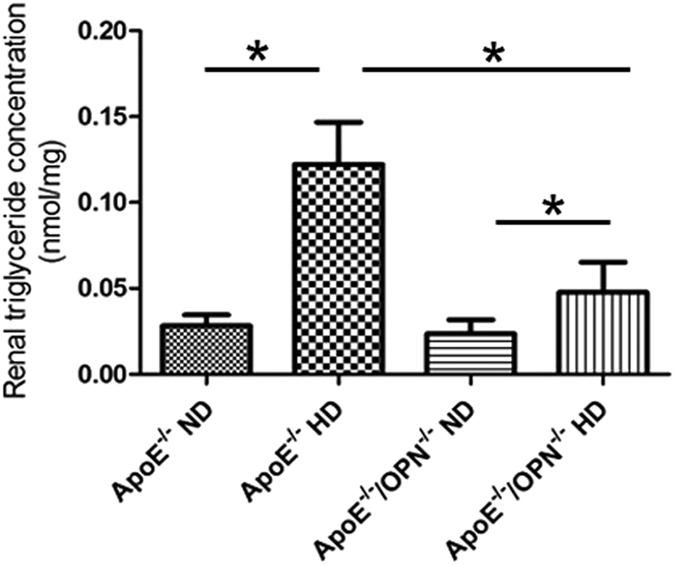
Renal triglyceride concentration in the four groups after 4 weeks of dietary treatment. Triglyceride concentration was measured in kidney tissue. Data are means ± SEM; n = 4 in each group. *P < 0.05. Two-way ANOVA, gene, p < 0.05; diet, p < 0.05; gene × diet, p < 0.05.

To confirm the decreased lipid accumulation in ApoE^*−/−*^/OPN^*−/−*^HD mice compared with ApoE^*−/−*^HD mouse, we measured the triglyceride concentration in kidney tissue. In agreement with oil red O staining results, ApoE^*−/−*^/OPN^*−/−*^ mice showed markedly reduced renal triglyceride concentration compared with ApoE^*−/−*^ mice despite consumption of HD (Fig. 3). Two-way ANOVA showed the effects of gene and diet on the renal triglyceride concentration (gene, p < 0.05; diet, p < 0.05; gene × diet, p < 0.05). Post-hoc analysis leveled that renal triglyceride concentration was reduced in ApoE^*−/−*^/OPN^*−/−*^HD mice compared with ApoE^*−/−*^HD mice (p < 0.05).

#### OPN deficiency reduced foam cell formation and glomerulosclerosis in high-cholesterol diet-fed mice

**Figure 4:**
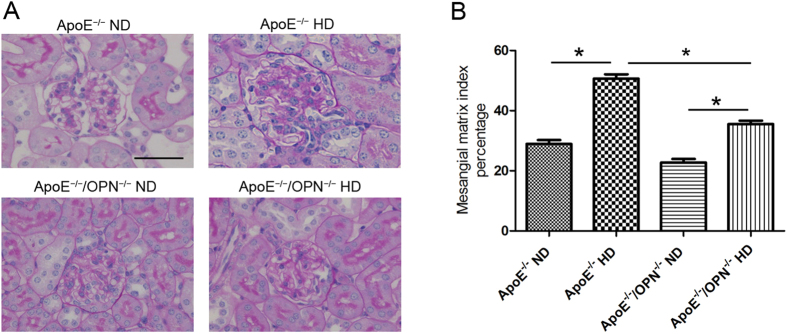
Glomerular mesangial area of the four groups after 4 weeks of dietary treatment. (**A**) Representative histological photomicrograph of glomeruli by periodic acid–Schiff (PAS) staining. Scale bar = 50 μm. (**B**) Bar graph summarizing the mesangial matrix index. Data are means ± SEM; n = 4 in each group. *P < 0.01. Two-way ANOVA, gene, p < 0.001; diet, p < 0.001; gene × diet, p < 0.001.

Figure 4A shows representative histological changes in each treatment group. Periodic acid-Schiff (PAS)-positive area with foam cells was observed in the mesangial region, and this was enlarged in the HD groups, especially in ApoE^*−/−*^HD mice. Glomerular volume and mesangial area were quantified by scanning glomeruli of each kidney section stained by PAS. Mesangial matrix index was calculated as mesangial area divided by glomerular area. Two-way ANOVA analyses showed that gene and diet significantly effect on the mesangial matrix index in glomerular (gene, p < 0.001; diet, p < 0.001; gene × diet, p < 0.001). Post-hoc analysis indicated that the mesangial matrix index was increased in ApoE^*−/−*^HD mice compared with ApoE^*−/−*^ND, but this effect was significantly suppressed in ApoE^*−/−*^/OPN^*−/−*^HD mice (Fig. 4B).

**Figure 5:**
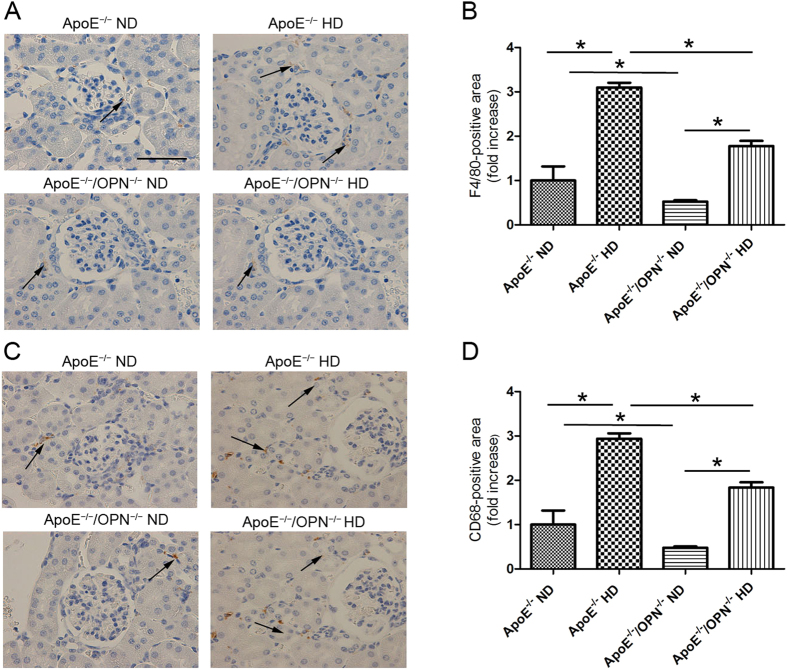
Macrophage content in the four groups after 4 weeks of dietary treatment. Representative immunohistochemistry for F4/80 (**A**) and CD68 (**C**) in the kidney. Scale bar = 50 μm. Arrows indicate positive staining cells. Bar graph summarizing the F4/80 (**B**) and CD68 (**D**)-positive cells in interstitial lesions. Data are means ± SEM; n = 4 in each group. *P < 0.01. Two-way ANOVA, gene, p < 0.001; diet, p < 0.001; gene × diet, p < 0.05.

**Figure 6:**
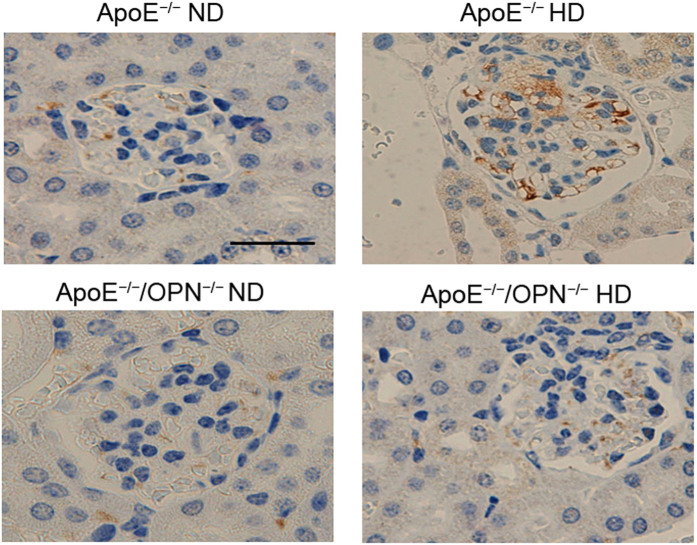
Megsin expression in the glomeruli of the four groups after 4 weeks of dietary treatment. Representative immunohistochemistry for megsin in the glomeruli. Scale bar = 50 μm.

To detect infiltrating macrophages, immunohistochemical analysis was performed using F4/80 and CD68 staining. F4/80 and CD68-positive cells were detected in interstitial lesions but not in the mesangial regions in glomeruli of ApoE^*−/−*^HD mice and ApoE^*−/−*^/OPN^*−/−*^HD mice. Only a few infiltrating F4/80 and CD68-positive cells were detected in the normal diet treatment groups (Fig. 5A,C). Two-way ANOVA showed the effects of gene and diet on F4/80-positive cells (gene, p < 0.001; diet, p < 0.001; gene × diet, p < 0.05) and CD68-positive cells (gene, p < 0.001; diet, p < 0.001; gene × diet, p < 0.05) which were detected in interstitial lesions. Post-hoc analysis leveled that infiltrating F4/80 and CD68-positive cells detected in ApoE^*−/−*^HD mice were reduced in ApoE^*−/−*^/OPN^*−/−*^HD mice (p < 0.001 and p < 0.001, respectively) (Fig. 5B,D). These results indicate that foam cells in mesangial lesions originate from mesangial cells. To conform this, megsin (predominantly expressed in mesangial cells) staining was performed (Fig. 6). Mesangial cells, especially foam cells were positive stained by anti-megsin antibody. These result conformed origin of the foam cells was mesangial cell.

**Figure 7:**
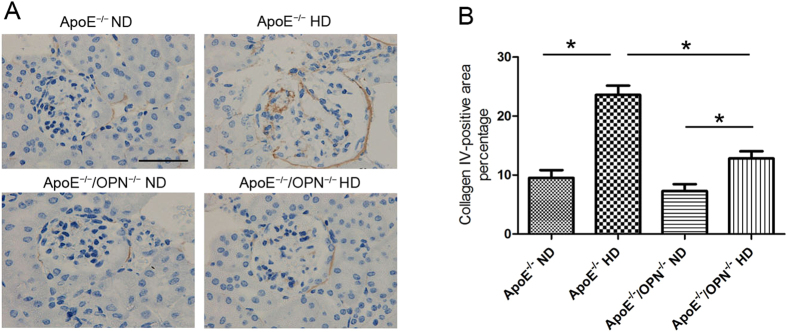
Collagen type IV expression in the glomeruli of the four groups after 4 weeks of dietary treatment. (**A**) Representative immunohistochemistry for collagen type IV in glomeruli. Scale bar = 50 μm. (**B**) Bar graph summarizing collagen type IV expression in glomeruli. Each bar represents the mean ± SEM; n = 4 in each group. *P < 0.001. Two-way ANOVA, gene, p < 0.001; diet, p < 0.001; gene × diet, p < 0.001.

To evaluate glomerulosclerosis, collagen type IV immunostaining was performed (Fig. 7A). Two-way ANOVA analyses showed that gene and diet significantly effect on the collagen type IV expression (gene, p < 0.001; diet, p < 0.001; gene × diet, p < 0.001). Post-hoc analysis showed that in ApoE^*−/−*^/OPN^*−/−*^ mice, collagen type IV accumulation in glomeruli were markedly reduced compared with that in ApoE^*−/−*^ mice consuming HD (Fig. 7B). This result indicates that OPN deficiency reduced glomerulosclerosis in ApoE^*−/−*^HD mice.

#### OPN deficiency reduced LOX-1 gene expression in the glomeruli of ApoE^*−/−*^ mice with HD

To investigate the mechanism of lipid accumulation in glomeruli, glomerular gene expression of relevant receptors, including LOX-1, scavenger receptor-class A (SR-A), scavenger receptor class B (CD36), low-density lipoprotein receptor (LDL-r) and the ATP-binding cassette transporter A1 (ABCA1) were examined by real time RT-PCR. Two-way ANOVA showed the effects of gene and diet on the glomerular gene expression of relevant receptors, LOX-1(gene, p < 0.001; diet, p < 0.001; gene × diet, p = 0.0931), SR-A (gene, p = 0.816; diet, p < 0.001; gene × diet, p < 0.05), CD36 (gene, p = 0.9317; diet, p < 0.05; gene × diet, p = 0.1741), ABCA1 (gene, p = 0.3797; diet, p < 0.001; gene × diet, p = 0.4575), LDL-r (gene, p = 0.0805; diet, p = 0.2705; gene × diet, p = 0.4816). Post-hoc analysis leveled that glomerular LOX-1 gene expression in ApoE^*−/−*^HD mice was reduced in ApoE^*−/−*^/OPN^*−/−*^HD mice (p < 0.001).

**Figure 8:**
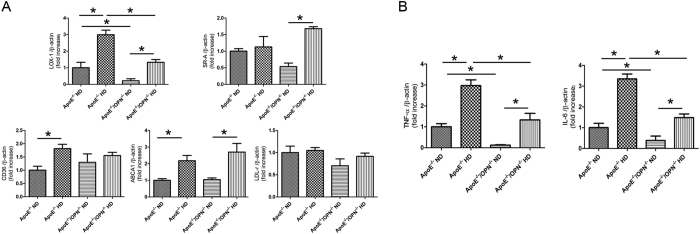
LDL and scavenger receptor and pro-inflammatory gene expression in the glomeruli of the four groups after 4weeks of dietary treatment. (**A**) Relative mRNA expression of LOX-1, SRA, CD36, LDL-r, and ABCA1 in the glomeruli of each group after 4 weeks of dietary treatment. (**B**) Relative mRNA expression of IL-6 and TNF-α in the glomeruli of each groups after 4weeks of dietary treatment. Data are means ± SEM; n = 4–6 in each group.*P < 0.001. Two-way ANOVA, LOX-1(gene, p < 0.001; diet, p < 0.001; gene × diet, p = 0.0931), SR-A (gene, p = 0.816; diet, p < 0.001; gene × diet, p < 0.05),CD36 (gene, p = 0.9317; diet, p < 0.05; gene × diet, p = 0.1741), ABCA1 (gene, p = 0.3797; diet, p < 0.001; gene × diet, p = 0.4575), LDL-r (gene, p = 0.0805; diet, p = 0.2705; gene × diet, p = 0.4816), IL-6 (gene, p < 0.001; diet, p < 0.001; gene × diet, p < 0.05), TNF-α (gene, p < 0.001; diet, p < 0.001; gene × diet, p = 0.1027).

Glomerular LOX-1 gene expression in ApoE^*−/−*^/OPN^*−/−*^ND mice was significantly lower than in ApoE^*−/−*^ND mice. HD significantly increased LOX-1 gene expression both in glomeruli of ApoE^*−/−*^HD and ApoE^*−/−*^/OPN^*−/−*^ mice, however, the expression levels of LOX-1 gene were similar between ApoE^*−/−*^ND and ApoE^*−/−*^/OPN^*−/−*^HD mice. Expression of CD36 and ABCA1 were increased in ApoE^*−/−*^HD mice compared with ApoE^*−/−*^ND mice, however, levels were similar to those of ApoE^*−/−*^/OPN^*−/−*^HD mice. Expression of other receptors, including SR-A, and LDL-r did not differ between ApoE^*−/−*^HD mice and ApoE^*−/−*^/OPN^*−/−*^HD mice (Fig. 8A). These results suggest that LOX-1, CD36 and ABCA1 influence lipid accumulation in the glomeruli of ApoE^*−/−*^HD mice. LOX-1, in particular, appears to be a critical factor for mitigation of lipid accumulation in the glomeruli of ApoE^*−/−*^/OPN^*−/−*^HD mice compared to ApoE^*−/−*^HD mice.

#### OPN deficiency reduced tumor necrosis factor (TNF)-α and interleukin (IL)-6 gene expression in the glomeruli of ApoE^*−/−*^ mice fed HD

To examine the involvement of pro-inflammatory cytokines in hypercholesteremic kidney disease, glomerular IL-6 and TNF-α gene expression were measured by real-time PCR. Two-way ANOVA showed the effects of gene and diet on the glomerular gene expression of relevant receptors, IL-6 (gene, p < 0.001; diet, p < 0.001; gene × diet, p < 0.05), TNF-1 (gene, p < 0.001; diet, p < 0.001; gene × diet, p = 0.1027). Post-hoc analysis leveled that IL-6 (p < 0.001) and TNF-α (p < 0.001) gene expression significantly increased in glomeruli of ApoE^*−/−*^HD mice compared with those of ApoE^*−/−*^ ND mice. Both IL-6 (p < 0.001) and TNF-α (p < 0.001) were upregulated in ApoE^*−/−*^HD mice; however this up-regulation was attenuated in ApoE^*−/−*^/OPN^*−/−*^HD mice (Fig. 8B).

### *In vitro* studies

#### OPN upregulates LOX-1 gene expression in primary mesangial cells

**Figure 9:**
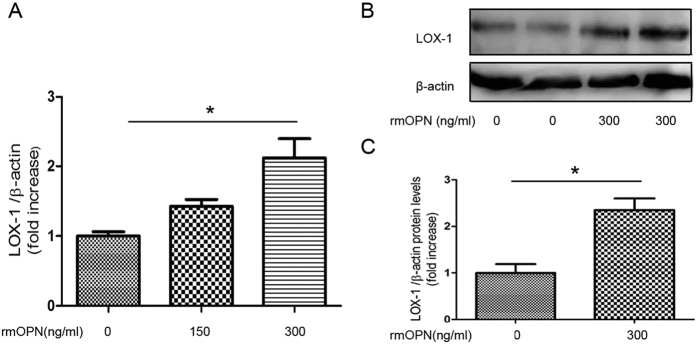
Recombinant mouse OPN(rmOPN) induces LOX-1 expression in primary mesangial cells. (**A**) Relative mRNA expression of LOX-1 in primary mesangial cells. Primary mesangial cells were incubated with rmOPN (0 ng/mL, 150 ng/mL, 300 ng/mL) for up to 24 h. (**B**) Immunoblotting for LOX-1 protein in primary mesangial cells. Primary mesangial cells were incubated with or without rmOPN (0 ng/mL, 300 ng/mL) for up to 48 h. (**C**) Bar graph shows quantification of LOX-1 protein expression. Data are means ± SEM; n = 4–6 in each group. Difference between two groups was evaluated by Student’s t-test for unpaired variables and differences among three groups were analyzed by one-way ANOVA and subsequent Tukey’s test. *P < 0.05.

To investigate the relationship between OPN and LOX-1, cultured primary mesangial cells were incubated with rmOPN for up to 24 h. There was a dose-dependent upregulation of LOX-1 expression with rmOPN (Fig. 9A). Forty eight hours incubation with rmOPN increased LOX-1 protein expression (Fig. 9B,C). The results indicated that OPN directly up-regulates LOX-1 expression in mesangial cells.

#### OPN stimulates ERK signal transduction in primary mesangial cells

**Figure 10:**
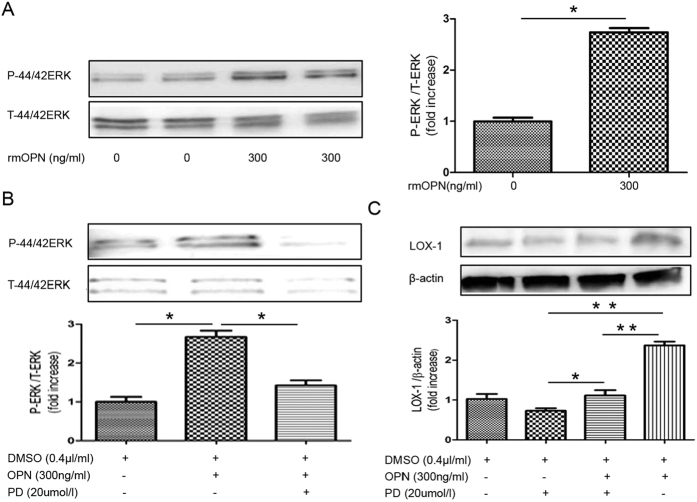
Recombinant OPN stimulates ERK signaling in primary mesangial cells. (**A**) Immunoblot for phosphorylated ERK and total ERK. Primary mesangial cells were incubated with rmOPN (0 ng/ml, 300 ng/mL) for up to 30 min. Bar graph shows quantification of phosphorylated ERK and total ERK expression. (**B**) Immunoblot for phosphorylated ERK and total ERK following pre-treatment with an ERK inhibitor. Primary mesangial cells were grown in culture and pre-incubated with an inhibitor of ERK (PD98059; 20 μmol/L) for 30 min followed by treatment with rmOPN for 30 min. Bar graph shows quantification of phosphorylated ERK and total ERK expression. (**C**) Immunoblot for LOX-1. Primary mesangial cells were pre-incubated with an inhibitor of ERK (PD98059; 20 μmol/L) for 30 min followed by treatment with rmOPN for 48 h. Bar graph shows quantification of LOX-1 protein expression. RmOPN induced LOX-1 protein expression in primary mesangial cells. Data are means ± SEM; n = 3 in each group. Difference between two groups was evaluated by Student’s t-test for unpaired variables and differences among three and four groups were analyzed by one-way ANOVA and subsequent Tukey’s test. *P < 0.05; **P < 0.01.

Next, we examined the involvement of the ERK pathway in LOX-1 upregulation. ERK phosphorylation was induced by 300 ng/mL rmOPN treatment of primary mesangial cells (Fig. 10A). Pre-incubation with an ERK inhibitor (20 μmol/L PD98059) for 30 min, followed by treatment with rmOPN for 30 min, reduced ERK phosphorylation (Fig. 10B) and LOX-1 upregulation (Fig. 10C).

## Discussion

This study demonstrates that OPN deficiency has a protective effect against the progressive lipid deposition and glomerulosclerosis elicited by hypercholesterolemia. In the bloodstream of humans and other vertebrates, cholesterol is transported in lipoprotein complexes. ApoE binds cholesterol for transport through the circulatory system as ApoE-containing chylomicrons and very-low-density lipoprotein (VLDL) particles. ApoE^*−/−*^ mice significantly show elevated serum cholesterol levels and were widely used as a model to study hypercholesterolemia^1^.

According to the metabolic characteristics, we found that TC and LDL-C were increased and TG decreased in ApoE^*−/−*^HD mice compared with ApoE^*−/−*^ND mice. Those results were agreement with Daniel Kolbus reports^14^. Interestingly, TC and LDL-C in ApoE^*−/−*^ mice were lower than in ApoE^*−/−*^/OPN^*−/−*^ mice with both ND and HD. These results indicated that OPN influences the cholesterol metabolisms, however, further studies are needed to clarify the mechanisms.

In spite of pathological changes by high cholesterol, BUN did not differ among the four groups. It may be due to the short duration of treatment with high cholesterol diet. In other words, morphological changes by lipid accumulation were not so terrible to increase BUN.

Hyperlipidemia promotes glomerular lipid deposition, increased mesangial matrix area and cellularity and glomerulosclerosis^15^,^16^,^17^. Cellular lipid homeostasis involves regulation of the influx, synthesis, catabolism, and efflux of lipids. An imbalance in these processes can result in conversion of macrophages, mesangial cells, and vascular smooth muscle cells into foam cells. This process is mediated by several independent pathways, including SR-A, class B (CD36), and LOX-1, whereas cholesterol efflux is primarily mediated by liver X receptor α/β (LXRα/β), which serves as an intracellular cholesterol sensor and regulates expression of its target gene ABCA1^18^,^19^,^20^. In the present study, we analyzed gene expression of LDL receptor and scavenger receptors including SR-A, CD36, and LOX-1. In these receptors, LOX-1, in particular, appears to be a critical factor for mitigation of lipid accumulation in the glomeruli of ApoE^*−/−*^/OPN^*−/−*^HD mice compared to ApoE^*−/−*^HD mice, because glomerular LOX-1 gene expressions were lower in ApoE^*−/−*^/OPN^*−/−*^ND and ApoE^*−/−*^/OPN^*−/−*^HD than in ApoE^*−/−*^ND and ApoE^*−/−*^HD mice, respectively.

LOX-1 was originally identified in endothelial cells, and is a 50-kDa type II membrane glycoprotein that contains a short N-terminal cytoplasmic domain, a single transmembrane domain, a short neck or stalk region, and an ox-LDL-binding C-terminal extracellular C-type lectin-like domain. On the cell surface, LOX-1 is comprised of 3 homodimers bound to ox-LDL, and plays a leading role in ox-LDL uptake and foam cell formation^21^,^22^. In contrast, deletion of LOX-1 reduced uptake of oxidized LDL and inhibited atherosclerosis in high-cholesterol diet-fed mice^23^. Thus, suppression of LOX-1 expression in ApoE^*−/−*^/OPN^*−/−*^HD mice may reduce foam cell formation. The downstream pathways activated by binding of oxidized LDL to LOX-1 not only contribute to lipid accumulation, but also trigger metabolic events that influence important biological functions that occur with atherosclerosis. Previous reports indicated that LOX-1 is upregulated by oxidized LDL, angiotensin II, pro-inflammatory cytokines, and shear stress^24^,^25^,^26^,^27^. The present results indicate that LOX-1 gene expression is regulated by one particular pro-inflammatory cytokine, OPN. To confirm the involvement of OPN in LOX-1 gene regulation, we performed an *in vitro* study and showed that OPN induced LOX-1 gene and protein up-regulation in primary mesangial cells. Li *et al*. have reported that glucose enhances macrophage LOX-1 expression. This enhanced LOX-1 expression was attenuated by inhibitors of PKC, ERK, and NF-κB, indicating that increased production of intracellular ROS and activation of the PKC/MAPK pathways are initial signaling events in the regulation of LOX-1 gene^28^. Pro-inflammatory genes (TNF-α and IL-6) were reported to be expressed at high levels, and to contribute to kidney injury in hyperlipidemia^29^,^30^,^31^. In contrast, TNF-α and IL-6 were shown to induce LOX-1 upregulation in smooth muscle cells^27^. The present study shows that TNF-α and IL-6 gene expression is reduced in ApoE^*−/−*^/OPN^*−/−*^HD mice compared with ApoE^*−/−*^HD mice. Attenuation of TNF-α and IL-6 expression may also reduce LOX-1 expression in ApoE^*−/−*^/OPN^*−/−*^HD mice.

Previously Nicholas *et al*. reported that OPN activated the ERK and JNK signaling pathways, which are known to stimulate TGF-β production in mouse mesangial cells (MES 13)^32^. Our *in vitro* study showed that OPN directly induced LOX-1 expression and this induction was attenuated by an ERK inhibitor, indicating that OPN activates the ERK signaling pathway, which stimulates LOX-1 in primary mesangial cells.

However, HD could significantly up-regulate LOX-1 gene expression in ApoE^*−/−*^/OPN^*−/−*^HD mice to the similar levels in ApoE^*−/−*^ND mice. These results suggested that another mechanism exists influencing LOX-1 gene expression beside OPN.

We also found that glomerular CD36 gene expression was significantly increased in ApoE^*−/−*^HD mice compared with ApoE^*−/−*^ND mice, however, in ApoE^*−/−*^/OPN^*−/−*^mice, HD did not influence CD36 gene expression. This failure may be contributed to the diminished accumulate lipid in ApoE^*−/−*^/OPN^*−/−*^HD mice.

Glomerular injury induced by hyperlipidemia is usually associated with an increased number of glomerular macrophages. The mechanism of macrophage accumulationis not fully understood, however, oxidized lipoproteins themselves can promote monocyte chemotaxis through the induction of specific chemoattractants^33^,^34^,^35^. Mesangial cells also oxidize lipoproteins and endocytose these modified lipoproteins through scavenger receptors^36^,^37^,^38^. The markers F4/80 and CD68 identified two populations of macrophages, CD68 and F4/80 positive cells were especially seen infiltrating the glomeruli and interstitial lesion, respectively^15^,^39^. In present study, immunohistochemical staining with anti-F4/80 and CD68 antibody did not find any obvious macrophages in glomeruli both ApoE^*−/−*^HD and ApoE^*−/−*^/OPN^*−/−*^HD mice. Megsin as a marker was widely used for mesangial cells expression. Megsin expression was reported to be increased in glomeruli of diabetic mice compared with nondiabetic mice^40^. Our results showed that foam cells in the glomeruli were positively stained with anti-megsin antibody markedly in ApoE^*−/−*^HD mice. Those results indicating that the foam cells are derived from mesangial cells. Tomiyama-Hanayama *et al*. also reported that ApoE^*−/−*^ mice fed a high-fat diet for 4 weeks showed striking lipid deposition and foam cell formation in glomeruli. However, only a few macrophages were detected in the glomeruli of ApoE^*−/−*^ mouse in that study^31^. The limited infiltration of macrophages into glomeruli may be due to the short time frame of high-cholesterol diet administration in our experiment. A similar phenomenon was recently reported in studies of a bovine growth hormone (bGH) mouse model of progressive glomerular sclerosis. Transgenic mice that over express bGH showed a significant increase in glomerular lipid content. Accumulation of intracellular lipids in the renal cortices of bGH mice was detectable at 5 weeks of age. However, CD68 expression, a marker for macrophage infiltration, was increased at 12 weeks but not at 5 weeks of age, suggesting that glomerular lipid accumulation can occur without macrophage infiltration^41^.

Hyperlipidemia has been shown to accelerate the induction and progression of renal injury leading to glomerulosclerosis^17^,^42^,^43^. Glomerulosclerosis can be evaluated by measuring the accumulation of collagen IV as detected by immunohistochemistry^44^,^45^. In our study, collagen type IV staining was significantly suppressed in ApoE^*−/−*^/OPN^*−/−*^HD mice compared with ApoE^*−/−*^HD mice, indicating that osteopontin deficiency contributes to reduced glomerulosclerosis and lipid accumulation. This confirms our previous report, which showed that OPN deficiency reduced aldosterone-induced kidney injury as indicated by suppression of collagen type IV accumulation^13^.

The limitations of our study are as follows. 1. ApoE^*−/−*^ mouse shows extremely high concentration of LDL-C compared with human patients with high LDL-C and so this model is not applied as a generalized human high LDL-C patient. 2. The duration of the treatment of high cholesterol diet in our study was only 4 weeks, because longer than 4 weeks treatment of high cholesterol diets induces high mortality. 3. Inbred, isolation colonies of ApoE^*−/−*^ mouse and ApoE^*−/−*^/OPN^*−/−*^ mouse may differ substantially with respect to background mutations and microbiota difference. 4. Present study indicated that a large number of foam cells in glomeruli were originated from mesangial cells, however, we could not deny the possibility that a few foam cells are originated from infiltrated macrophages. 5. Finally, TC and LDL-C were slightly but significantly lower in ApoE^*−/−*^/OPN^*−/−*^ mice than those in ApoE^*−/−*^ mice with both diets. These differences would influence the pathological changes in the kidneys.

In conclusion, our data establish that OPN deficiency contributes to mitigation of hypercholesteremic kidney injury as shown by downregulation of LOX-1, and suppression of foam cell formation, lipid deposition, and glomerulosclerosis. These findings provide new insights into the role of OPN in hypercholesterolemic kidney injury and raise the possibility of a novel therapeutic intervention for the progression of chronic kidney disease.

## Materials and Methods

### Animals and experimental protocols

All animal studies were approved by the Animal Studies Committee of Ehime University. ApoE^*−/−*^ (B6.129P2-*Apoe*^*tm1Unc*^/J) mice were purchased from Jackson Laboratory (Bar Harbor, ME, USA). OPN^*−/−*^ mice with a targeted mutagenesis of the secreted phosphoprotein 1 (*spp-1*) were generated as described previously^46^ and ApoE^*−/−*^OPN^*−/−*^ with the same C57BL/6J genetic background were generated by crossing ApoE^*−/−*^and OPN^*−/−*^ mice. Both ApoE^*−/−*^ and ApoE^*−/−*^OPN^*−/−*^ mice were generated from isolated inbred stocks. OPN genotyping was performed by the polymerase chain reaction analysis of tail DNA. All mice were housed in a room with a 12:12 h light-dark cycle, with room temperature maintained at 24 °C. At 8 weeks old, the mice were randomly divided into four groups: ApoE^*−/−*^ mice fed normal diet (n = 8) or high-cholesterol diet (n = 8), and ApoE^*−/−*^/OPN^*−/−*^ mice fed normal diet (n = 8) or high-cholesterol diet (n = 8). The normal diet contained 5.3% fat, 23.6% protein and 2.9% crude fiber (Oriental Yeast Co, Ltd., Tokyo, Japan) and the high-cholesterol diet contained 1% cholesterol, 15% fat, 23.6% protein and 2.9% crude fiber (Oriental Yeast Co, Ltd.). Each group was fed their diet for 4 weeks. Blood samples were obtained from the inferior vena cava and collected in serum tubes and stored at −80 °C until used. Coronal sections of the kidneys were fixed in 10% formalin and then embedded in paraffin for histological evaluation or embedded in OCT Compound (Torrance, CA, USA) and stored at −80 °C for oil-red O staining. The remainder of the kidney was snap-frozen in liquid nitrogen for mRNA or immunohistochemical analysis. All animal experiments were performed in accordance with the Guide for the Care and Use of Laboratory Animals. The study was approved by the ethical committee of Ehime University Graduate School of Medicine.

### Biochemical measurements

TC, LDL-C, BUN and TG were measured using an automatic analyzer (Nagahama LSL, Shiga, Japan). Plasma OPN was measured using commercially available ELISA kits (IBL, Gunma, Japan).

### Renal triglyceride assay

For triglyceride assay from kidney tissue, lipid was extracted from homogenized kidney by the Folch method^47^. Triglyceride concentration was determined using a triglyceride quantification kit (BioVision, San Francisco, USA) and detected using a Versa Maxmicroplate reader (Molecular Devices, Sunnyvale, USA). Concentrations were determined using a Versa Maxmicroplate reader (Molecular Devices). Assays were performed in duplicate.

### Morphologic analysis and immunohistochemistry

Kidney sections were stained using the PAS method. Paraffin-embedded histological sections cut at 4 μm thickness were taken after series of alcohol (70% to 100%) washings and deparaffinization. After deparaffinization and hydration, sections were placed in 1% periodic acid for 15 min followed by water wash and Schiff’s reagent (Sigma-Aldrich, St. Louis, MO, USA) treatment, followed by staining with Gills hematoxylin (Thermo, MA, USA). Cross sections of PAS staining were semi-quantified using NIH Image J software (http://rsb.info.nih.gov/ij/). Glomerular volume and mesangial area were quantified by scanning 10 non-overlapping glomeruli in each kidney section stained by PAS. Mesangial matrix index was calculated by the expressing the PAS positive area as a percentage of total glomerular area. Lipid accumulation was evaluated by oil red O staining. Frozen kidneys were cut into 6 μm sections with are frigerated microtome (LEICA CM 1100, Tokyo, Japan) and attached to SuperFrost plus slides (Thermo), air dried for up to 90 minutes at room temperature, and then stained with oil red O dye (Sigma-Aldrich) to detect the presence of lipids. Sections were washed three times in PBS and fixed in calcium formal: 4% formaldehyde and 1% calcium chloride for 1 hour at room temperature. Next, samples were incubated for 15 minutes in 60% isopropanol and then stained for 15 minutes with oil red O solution. Samples were then briefly rinsed in 60% isopropanol, washed thoroughly in water, counterstained in Mayers Hematoxylin solution (Thermo) and mounted under coverslips in Glycergel. Glomerular volume and lipid accumulation were quantified by scanning 10 non-overlapping glomeruli from each kidney section with Image J software and expression the oil red O positive area as a percentage of the total area.

Immunohistochemistry was performed using Histone Simple stain kits (Nichirei, Tokyo, Japan) according to the manufacturer’s instructions. Briefly, paraffin-embedded sections were deparaffinized with xylene and then rehydrated in a descending series of ethanol washes. The sections were treated for 15 min with 3% H_2_O_2_ in methanol to inactivate endogenous peroxidases and then incubated at room temperature for 1 hour with primary antibodies to collagen IV (rabbit anti-collagen IV antibody, 1:500; Abcam, England), F4/80 (rat anti-F4/80 antibody, 1:500; Abcam), CD68 (rabbit anti-CD68 antibody, 1:500; Abcam), megsin (rabbit anti-megsin antibody, 1:50; Santa, Cruz, CA, USA). Positive staining with diaminobenzidine was quantified using Image J software by scanning 10 non-overlapping fields of each kidney section, and expressing the positive area as a percentage of the total area.

### Isolating glomeruli

Mouse glomeruli were extracted as described previously^48^. All steps were performed in ice-cold PBS. Kidneys were perfused with cold PBS and harvested from mice. The cortex of each kidney was separated by macroscopic dissection with a sharp knife. The cortex tissue was carefully minced with the same knife on a pre-cooled glass dish in sterile PBS. The homogenized tissue was then pushed through a stainless sieve with a pore size of 90 μm by applying gentle pressure with a stencil. The sieve was rinsed several times with PBS. The tissue below the sieve, containing an enrichment of glomeruli, was collected and transferred to a sieve having a pore opening of 53 μm. After several washings with 50 mL PBS, the material that remained on top of the sieve, and which contained the glomeruli was collected in 50 mL of PBS and centrifuged for 8 min at 1200 r.p.m.The supernatant was decanted while the pellet containing the glomeruli was re-suspended in PBS. The washing step was repeated two to three times until the supernatant was clear. Isolated glomeruli were assessed by light microscopy of the final suspension.

### Cell culture

Primary mesangial cells (MCs)were isolated from 8-week-old C57BL/6 mice by a sieving method^49^ and cultured in RPMI 1640 containing 20% fetal calf serum (FBS), 100 IU/mL penicillin, 100 μg/mL streptomycin (Sigma Chemical, St. Louis, MO, USA), and insulin–transferrin–selenium (Invitrogen, USA). MCs were maintained at 37 °C in an atmosphere of air and 5% CO_2_. MCs were grown to 90% confluence, serum-starved (0.1% fetal bovine serum) for 24 h and treated with rmOPN (0–300 ng/mL; R&D Systems, Minneapolis, MN, USA) for up to 24–48 h. Cells were lysed for isolation of mRNA and protein and evaluation of LOX-1 expression by quantitative RT-PCR and western blotting.

### RNA isolation and real-time RT-PCR

Total RNA was isolated from glomeruli using the RNeasy Mini Kit (QIAGEN, Tokyo, Japan) according to the manufacturer’s protocol. Complementary DNA (cDNA) was synthesized from total RNA using a first-strand cDNA synthesis kit (SuperScript VILO cDNA Synthesis Kit; Life Technologies Carlsbad, CA, USA), according to the manufacturer’s protocol. Gene expression was analyzed quantitatively by real-time RT-PCR using fluorescent SYBR Green technology (Light Cycler; Roche Molecular Biochemicals). β-actin cDNA was amplified and quantitated in each cDNA preparation in order to normalize the relative amounts of the target genes. Primer sequences are listed in Table 2.

### Western blot analysis

Western blot analysis was performed using standard procedures^50^. The lysates were separated on Any kD Mini-PROTEAN TGX Precast Gels (Bio-Rad), followed by immunoblotting using a primary antibody to LOX-1 (rabbit anti-LOX-1 antibody, 1:250; Abcam), total Erk1(Rabbit anti-Erk, 1:1000; Cell Signaling Technology, USA), Phospho-Erk (Rabbit anti-phospho-Erk, 1:2000; Cell Signaling Technology), anti-β-actin (1:1000; Cell Signaling Technology). Membranes were then incubated with secondary antibody (anti-rabbitIg-G, 1:1000; Cell Signaling Technology). This analysis was carried out independently three times. Protein levels are expressed as protein/β-actin ratios to minimize loading differences. The relative signal intensity was quantified using NIH Image J software.

### Statistical analysis

Data were expressed as means ± SEM. All data were analyzed using Ekuseru-Toukei 2012 (Social Survey Research Information Co. Ltd., Tokyo, Japan). Any differences between two groups were evaluated by Student’s t-test for unpaired variables and those among three or four groups *in vitro* study were analyzed by one-way ANOVA and subsequent Tukey’s test. The gene (ApoE^*−/−*^ and ApoE^*−/−*^OPN^*−/−*^) and diet (ND and HD) effects on results were analyzed by two-way ANOVA and subsequent Bonferroni test. P values < 0.05 were considered statistically significant.

## Additional Information

**How to cite this article**: Pei, Z. *et al*. Osteopontin deficiency reduces kidney damage from hypercholesterolemia in Apolipoprotein E-deficient mice. *Sci. Rep.* **6**, 28882; doi: 10.1038/srep28882 (2016).

## Figures and Tables

**Table 1:** Metabolic data from the four groups after 4 weeks of dietary treatment.

	ApoE^-/-^ND	ApoE^-/-^HD	ApoE^-/-/^OPN^-/-^ND	ApoE^-/-/^OPN^-/-^HD	Significance (P value)		
	n = 6	n = 6	n = 6	n = 6	Gene	Diet	Gene x Diet
Body weight (g)	25.17 ± 0.31	23.17 ± 0.17	25.67 ± 0.33	23.83 ± 0.31	NS	< 0.05	NS
Kidney weight (mg)	183.33± 2.11	165 ± 3.41	183.33 ± 4.94	168.33 ± 4.77	NS	< 0.05	NS
*TC* (mg/dl)	458 ± 39.47	2065± 138.42	362.5 ± 34.12	1640 ± 189.64	< 0.05	< 0.01	NS
LDL-C (mg/dl)	107.17 ± 12.1	519.17 ± 25.4	77.83 ± 5.32	414.17 ± 41.52	< 0.05	< 0.01	NS
*TG* (mg/dl)	119 ± 31.36	43.33 ± 9.89	92.5 ± 24.02	31.67 ± 3.07	NS	< 0.05	NS
BUN (mg/dl)	27.8 ± 2.49	29.58 ± 1.65	23.35 ± 1.75	27.25 ± 1.5	NS	NS	NS

Abbreviations: TC, total cholesterol; LDL-C, low-density lipoprotein- cholesterol; TG, triglycerides; BUN, blood urea nitrogen. Data are analyzed by two-way ANOVA; n = 6 per group. NS, not significant.

**Table 2:** Primer oligonucleotide sequences.

	Primers
LOX-1	F:5′-CAAAGTCTCCCAACCAACCTGCAA-3′
	R:5′-ACATCCTGTCTTTCATGCGGCAAC-3′
SRA1	F:5′-GTTAAAGGTGATGGGGGACA-3
	R:5′-TCCCCTTCTCTCCCTTTTGT-3′
CD36	F:5′-CCTTAAAGGAATCCCCGTGT-3′
	R:5′-TGCATTTGCCAATGTCTAGC-3′
LDL-r	F:5′-TTGGGTTGATTCCAAACTCCAT-3′
	R:5′-CCGATTGCCCCCATTGA-3′
ABCA1	F:5′-AGCCAGAAGGGAGTGTCAGA-3′
	R:5′-CATGCCATCTGGGTAAACCT-3′
TNF-α	F:5′-TCTCATGCACCACCATCAAGGACT-3′
	R:5′-ACCACTCTCCCTTTGCAGAACTCA-3′
IL-6	F:5′-TACCAGTTGCCTTCTTGGGACTGA-3′
	R:5′-TAAGCCTCCGACTTGTGAAGTGGT-3′
β-actin	F:5′-CGATGCCCTGAGGGTCTTT-3′
	R:5′-TGGATGCCACAGGATTCCAT-3′

Abbreviations: LOX-1, lectin-like oxidized low-density lipoprotein receptor-1; SRA, scavenger receptor-A; LDL-r, low-density lipoprotein receptor; ABCA1, ATP-binding cassette transporter A1; TNF-α, tumor necrosis factor-α; IL-6, interleukin-6.
